# Intrinsically chiral thermoresponsive assemblies from achiral clusters: enhanced luminescence and optical activity through tailor-made chiral additives†

**DOI:** 10.1039/d4sc07227h

**Published:** 2024-12-17

**Authors:** Camelia Dutta, Ragul Vivaz Nataraajan, Jatish Kumar

**Affiliations:** a Department of Chemistry, Indian Institute of Science Education and Research (IISER) Tirupati Andhra Pradesh 517619 India jatish@iisertirupati.ac.in

## Abstract

Chiral metal clusters, due to their intriguing optical properties and unique resemblance in size to biomolecules, have attracted a lot of attention in recent times as potential candidates for application in bio-detection and therapy. While several strategies are reported for the synthesis of optically active clusters, a facile approach that enhances a multitude of properties has remained a challenge. Herein, we report a simple strategy wherein the use of a chiral cationic surfactant, during the synthesis of achiral clusters, leads to the fabrication of chiral assemblies possessing enhanced luminescence and optical activity. The structural resemblance of the capping ligand, mercaptoundecanoic acid (MUA) to the co-surfactant, (+/−)-*N*-dodecyl-*N*-methylephedrinium bromide (DMEB), assisted specific interactions that led to the formation of chiral assemblies exhibiting unique thermoresponsive behavior and tunable optical activity. The symmetry breaking in the aggregates driven by the electrostatic interaction between the positively charged chiral surfactant and negatively charged clusters led to enhanced luminescence through aggregation induced enhanced emission (AIEE). The work highlighting the generation and tuning of cluster chirality provides newer insights into the fundamental understanding of nanocluster optical activity thereby opening newer avenues for the development of materials for application in the field of chiral biosensing and enantioselective catalysis.

## Abstract 2

Inherently chiral cluster aggregates were synthesized employing the chiral surfactant (+/−)-*N*-dodecyl-*N*-methylephedrinium bromide as a chiral dopant, yielding the mirror-image Cotton effect and thermoresponsive chiroptical properties.

## Graphical abstract

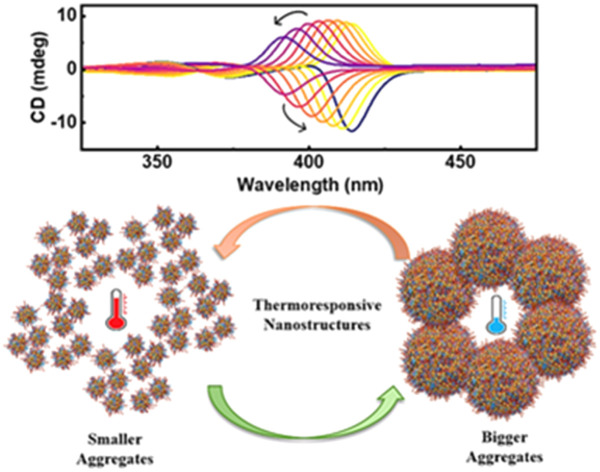

## Introduction

Atomically precise metal clusters, due to their intriguing physicochemical properties stemming from the quantum size effect, have captivated research interest over the years.^1–5^ Due to their exciting optical effects, these materials are finding potential applications in the field of optoelectronics, bioimaging, biosensing and data encryption.^6–10^ Most applications of these quantized particles revolve around their tunable luminescence properties.^11–13^ However, the luminescence quantum yield of most clusters remained relatively low compared to that of their organic fluorophores.^14–16^ Hence, research focus has been on developing strategies such as tailoring the surface ligands, doping dual metals and inducing cluster assembly, to improve the quantum efficiency.^17,18^ Among them, self-assembly leading to enhanced luminescence, termed as aggregation induced enhanced emission or AIEE, emulating similar effects observed in organic luminogens, has emerged as a promising technique.^19–22^ The success of AIEE in nanoclusters is largely dependent on the reproducibility and the robustness of the approach adopted for the aggregation of the clusters. The interest in self-assembled luminescent nanostructures is further intensified through the incorporation of symmetry breaking strategies that lead to the fabrication of chiral nanostructures.^23^

**Fig. 1:**
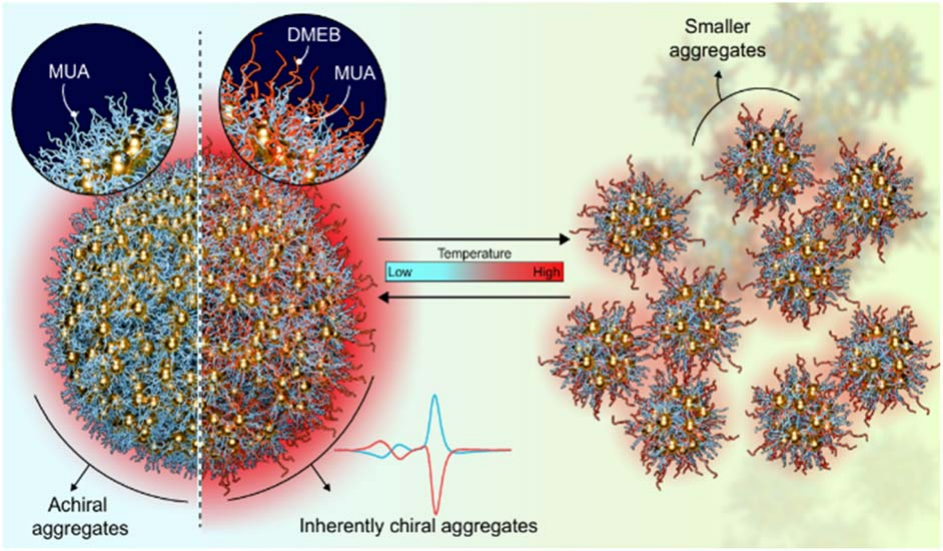
Scheme illustrating the formation of achiral and chiral luminescent cluster aggregates in the absence and presence of a chiral additive (DMEB), respectively. Their thermoresponsive characteristics upon temperature variation are also shown.

Optically active clusters form a very vital subgroup of coinage metal clusters.^24–27^ In addition to possessing all the fascinating properties of conventional metal clusters, this class of materials is supplemented with chirality as a unique attribute.^28^ Chirality is a common characteristic observed in nature at varying length scales ranging from sub atomic particles to large galaxies.^29–31^ However, the most important is the existence of unique chiral features in building blocks of living matter such as DNA and proteins. The resemblance of nanocluster size to the biological building blocks renders chiral clusters extremely important in several bio-applications such as sensing, imaging and therapy. Based on the mode of induction, chirality is rendered in metal clusters through one (or a combination) of three mechanisms: (i) formation of intrinsically chiral metallic cores, (ii) asymmetric arrangement on the achiral surface, or (iii) extrinsic chirality induced by peripheral ligands.^32–40^ Strategies developed for the synthesis of metal clusters may lead to enhanced performance of one of its characteristic properties, but not in a holistic manner. In this context, developing a facile approach that can combine the AIEE effect and the optical activity would result in clusters possessing a multitude of properties. Herein, we demonstrate the synthesis of 11-mercaptoundecanoic acid (MUA) capped negatively charged achiral gold clusters that exhibit enhanced luminescence through the AIEE effect. The mere presence of a small amount of chiral surfactant, namely, (+/−)-*N*-dodecyl-*N*-methylephedrinium bromide (DMEB), during the synthesis of clusters, assisted in the formation of chiral assemblies possessing enhanced optical activity and luminescence (Fig. 1). The unique structural features of chiral cationic surfactants such as (i) a long hydrocarbon chain resembling MUA, (ii) the quaternary ammonium group rendering surface charge, and (iii) a phenyl group along with chiral centers, played a significant role in the chiral self-organization of clusters in aqueous solution. The spectral changes observed upon varying the temperature and pH were utilized to enhance our understanding of the dynamics of self-assembly. While the excited state dynamics in such clusters have been probed earlier,^21^ there have been no attempts for understanding the dynamics in their optical activity. This endeavour holds significant promise for advancing our understanding of the chiral induction mechanism in metal clusters and unlocking new possibilities for their application in various fields.

## Results and discussion

**Fig. 2:**
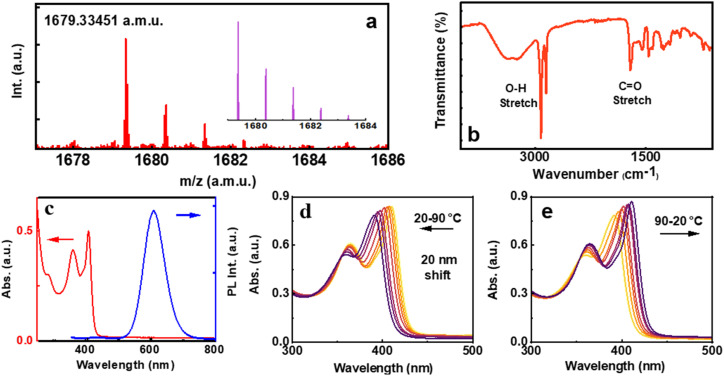
(a) MALDI-TOF and (b) FT-IR spectra of MUA capped Au clusters. Inset in ‘a’ shows the simulated mass spectrum. (c) Absorption (red trace) and emission (blue trace) spectra of the clusters in water. Absorption spectral changes during (d) gradual heating of clusters to 90 °C and (e) upon cooling back to room temperature.

The MUA capped Au clusters were synthesized following a reported method with modifications.^41^ After purification, the clusters were structurally characterized by MALDI-TOF, revealing a highly abundant species at a *m*/*z* of 1679.33 atomic mass units, corresponding to the formula of Na^+^ + Au_4_(C_11_H_21_SO_2_)_4_ (Fig. 2a and S1†). The experimental mass matches well with the simulated mass spectra confirming the sample purity (Fig. 2a, inset) and is in agreement with the reported structure of similar clusters.^21^ X-ray photoelectron spectroscopy (XPS) survey of Au 4f_7/2_ exhibits two intense peaks at a binding energy of around 83 and 86 eV corresponding to Au(0) and Au(i) of gold thiolate binding states, respectively (Fig. S2a†).^42^ FT-IR measurements helped to establish the binding nature of the metal and ligand. The absence of the thiol (–SH) stretching of the MUA ligand suggests the existence of well-established Au–S bonding. The stretching vibration of C=O is detected at 1721 cm^−1^, while the O–H stretch is observed in the range of 2800–2970 cm^−1^, suggesting that the carboxylic group remains intact upon formation of the cluster (Fig. 2b). TEM images reveal an average size less than 2 nm, confirming its classification as metal clusters (Fig. S3a†). However, the synthesis of clusters at a controlled pH resulted in distinct features, indicative of the self-assembly of the clusters as revealed by TEM images collected in the presence of the base (Fig. S3b†). These images showed the accumulation of clusters, suggesting their self-assembly, mostly influenced by the pH and alkyl chain length of the ligand, which is further investigated in detail.^43,44^

Photophysical characterization of the clusters carried out using UV-visible and photoluminescence spectroscopy revealed features characteristic of Au_4_ clusters. The UV-visible spectrum exhibited three distinct absorption peaks at 285, 363, and 410 nm, arising from the interband electronic transitions (Fig. 2c, red trace). The absence of a surface plasmon resonance (SPR) band in the 500–600 nm range indicates the absence of large Au nanoparticles in solution. The 285 nm peak originates from the electronic transitions between Au-5d and Au-6sp orbitals, involving metal-to-metal charge transfer (MMCT) and metal-to-ligand charge transfer (MLCT).^45^ On the other hand, the 410 nm peak arises from electronic transitions between s-3pπ and Au-6sp orbitals, involving ligand-to-metal charge transfer (LMCT) and ligand-to-ligand charge transfer (LLCT).^45^ The nanoclusters exhibited a strong red emission at 608 nm and a faint blue emission at 433 nm upon 285 nm excitation (Fig. 2c, blue trace). The luminescence quantum yield (QY) of the cluster was calculated to be 9.5%. The average photoluminescence lifetime of the emission centered at 608 nm was calculated to be 2.37 μs with a biexponential decay function (Fig. S4†). To our surprise, the cluster solution exhibited an interesting spectral shift with varying temperatures. A gradual blue shift in the major absorption peak was observed upon heating the sample to 90 °C. The spectral features could be fully regained upon cooling the solution back to 20 °C. The temperature-dependent absorption shift suggests that the aggregates in solution undergo a structural transformation upon heating, and revert back to the initial configuration when the system is cooled to room temperature (Fig. 2d and e). This phenomenon is examined in detail in the later part of the manuscript.

**Fig. 3:**
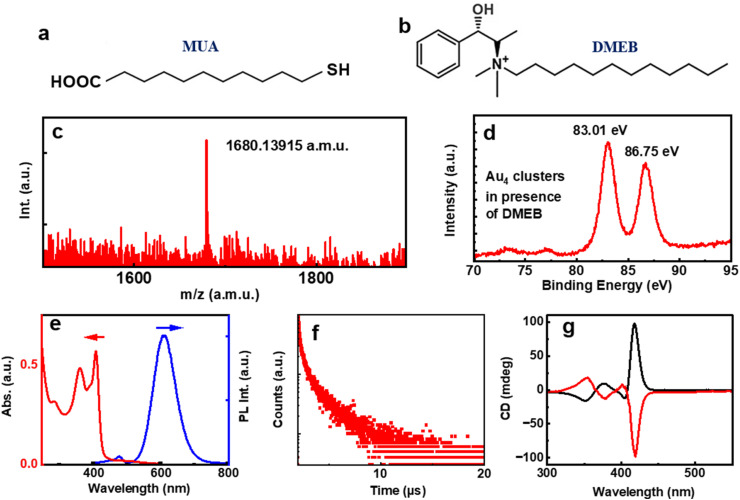
Structure of the (a) achiral ligand, MUA and (b) chiral additive, DMEB. (c) MALDI-TOF and (d) XPS plot of the clusters synthesized in presence of (−)-DMEB. (e) UV-visible absorption (red trace) and PL emission (blue trace), (f) PL lifetime decay and (g) CD spectra of the cluster solution. Red and black traces correspond to the CD signals obtained from the clusters in the presence of (+)- and (−)-DMEB, respectively.

The absence of any chiral agent rendered the Au_4_ clusters achiral (Fig. S5†). Our attempts were to design strategies to fabricate cluster aggregates possessing optical activity. To understand the surface charge of the synthesized Au_4_ clusters, the sample was subjected to zeta potential analysis. The clusters dispersed in aqueous solution exhibited a zeta potential value of −34.9 mV, may be due to the presence of carboxylic acid groups (Fig. S6a†). We attempted assembling the clusters using surfactants possessing positive charge. Due to its intriguing structural features (*vide supra*), DMEB was chosen as the chiral surfactant molecule. Varying amounts of DMEB were added to the Au_4_ clusters and spectral features were monitored. Unfortunately, no UV-visible or luminescence spectral changes were observed. The CD remained silent suggesting no noticeable interaction between the chiral molecule and the particles in solution and hence, ruling out the possibility of chiral induction into the Au_4_ clusters (Fig. S7†). Furthermore, we modified our synthetic strategy by introducing DMEB along with MUA during the synthesis of Au_4_ clusters intending to promote the electrostatic interaction between the surfactant and the Au_4_ units during its synthesis, leading to the formation of inherently chiral cluster aggregates possessing optical activity and the AIEE effect. The molar ratio of DMEB : MUA was maintained as low as 1 : 120. The MALDI-TOF spectra collected for the newly synthesized clusters showed similar peaks to pure MUA Au_4_ clusters (Fig. 3c), primarily eliminating the possibility of the formation of pure DMEB clusters or mixed ligand clusters. This observation was further supported by FT-IR measurements, where no thiol stretch was detected, ruling out the replacement of MUA ligands by DMEB (Fig. S8a†). Subsequently, the possibility of unbound DMEB was ruled out by comparing the FT-IR spectra of clusters with that of pure DMEB dispersed in water (Fig. S8b†). Fig. 3d presents the XPS plot of Au 4f where the oxidation state of the clusters remains similar. The zeta potential of the clusters synthesized in the presence of (+)/(−) DMEB was found to be −21.46 and −21.82 mV, respectively, a value much lower than that of pure MUA capped Au_4_ clusters, indicating a dynamic interaction between the clusters and DMEB (Fig. S6b and c†). Both the UV-visible and luminescence spectra of the nanostructures showed similar features to pure MUA capped Au_4_ clusters (Fig. 3e). The PL lifetime was found to be 2.08 μs for the clusters synthesized in the presence of DMEB (Fig. 3f). However, PLQY showed a substantial increment with a value of 19.70% in the presence of DMEB, indicative of an AIEE triggered by co-assembly of the clusters and DMEB. The assembly of the clusters was evidenced from both the cloudy appearance of the solution and the TEM images that showed irregular aggregates scattered on different parts of the grid (Fig. S9†).

**Fig. 4:**
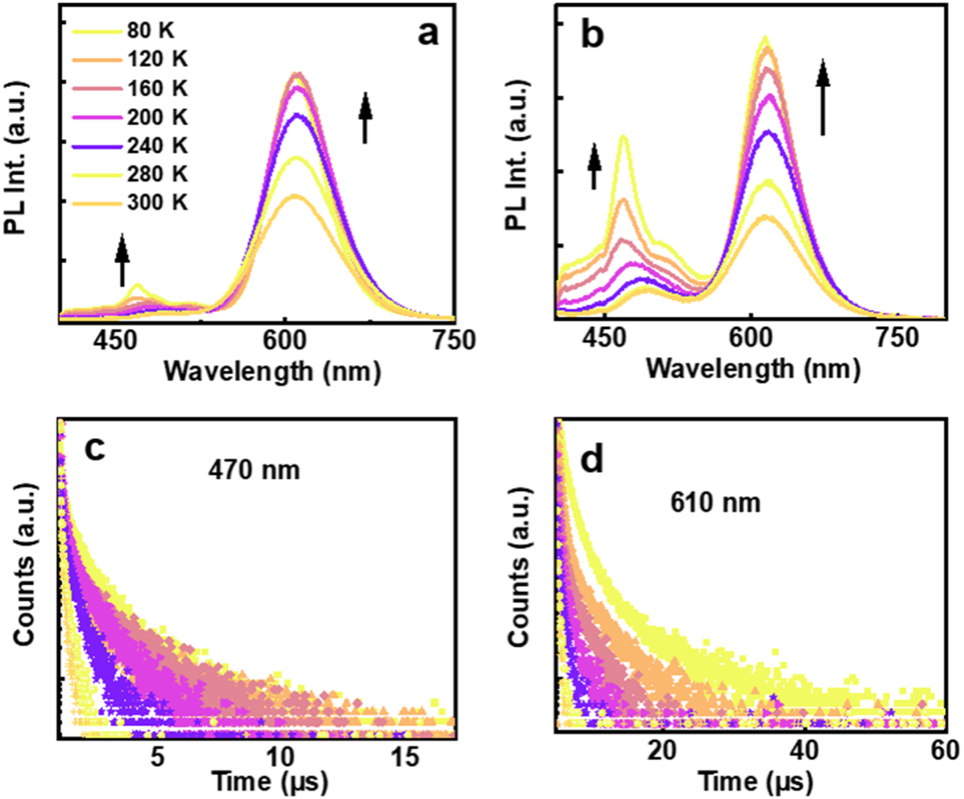
Temperature dependent PL spectra of MUA capped Au_4_ clusters in the (a) absence and (b) presence of the chiral surfactant DMEB. Temperature dependent lifetime plot of MUA capped Au_4_ clusters in the presence of DMEB for emission monitored at (c) 470 nm and (d) 610 nm.

To further understand the excited state dynamics of the aggregated clusters, lower temperature experiments were carried out on nanocluster incorporated self-standing transparent films fabricated using polyvinyl alcohol (PVA). Emission and lifetime measurements, ranging from 80 K to room temperature were carried out on films incorporated with clusters, both in the presence and absence of DMEB. The polymeric films displayed spectral features akin to their solution counterparts, with a slight shift in luminescence maxima (Fig. 4). At room temperature, lifetimes of 2.07 and 2.65 μs were observed for the red emission from films prepared in the presence and absence of DMEB, respectively, which were further enhanced to 8.43 and 8.49 μs, respectively, on lowering the temperature (Fig. 4d and S10b†). Conversely, for the blue emission, the lifetime values were 226.4 and 177.11 ns, respectively, for clusters synthesized in the presence and absence of DMEB, which were enhanced to 2.22 and 1.18 μs, respectively, at low temperature (Fig. 4c and S10a†). The enhancement in lifetime values may be attributed to the constrained vibrational motion in the film relative to that in solution. Hence, the long lifetime and the large Stokes shift observed at room temperature are suggestive of the involvement of the triplet state in the emission mechanism, and hence predominantly phosphorescence or thermally activated delayed fluorescence (TADF). Further exploration revealed the longer-lived excitons to be in equilibrium between the singlet and lower-lying triplet excited states (details in ESI, Fig. S11†). Similarly, a significant increase in lifetime observed in all the cases suggests the involvement of multiple energy states and transitions, which delays emission at lower temperatures, leading to significant enhancement in the lifetime values. Upon lowering the temperature, enhanced emission was observed in both cases (Fig. 4a and b). Interestingly, for the blue emission, multiple peaks were observed on lowering the temperature, further supporting the hypothesis on the formation of different types of aggregates in the presence of DMEB (Fig. 4b), which was not evident enough in the absence of DMEB (Fig. 4a).

Having established the assembly of the clusters in the presence of chiral surfactant DMEB, our interest was to probe their optical activity. Interestingly, the CD spectrum of the synthesized clusters showed intense peaks corresponding to its absorption in the UV and visible range. Mirror image spectra were observed for the clusters synthesized in the presence of opposite isomers of the chiral surfactant with negative and positive peaks at 413 nm for the clusters synthesized using (+) and (−) DMEB, respectively (Fig. 3g). A closer look at the CD signals show the presence of bisignate peaks with the crossover points at the absorption maxima, features typical of the exciton coupling model.^46,47^ This further confirms the coupling of the dipoles during the self-assembly of the clusters. The evolution of chirality was monitored over different reaction times and pH values to optimize the reaction conditions (Fig. S13 and S12†). A stirring time of 48 h was found to be optimal for obtaining intense CD signals (Fig. S13†). Experiments carried out at different pH values showed a loss of optical activity at acidic as well as basic pH and intense CD signals at an optimum pH (Fig. S12†). Hence, it can be concluded that the presence of DMEB at an optimal pH is the prerequisite for the co-assembly that can lead to chiral induction and enhanced QY through AIEE. Control experiments were further carried out to rule out the possibility of chirality originating from the interactions among DMEB, DMEB–MUA or MUA. The absence of emission at around 608 nm and CD or UV signals at around 260–440 nm confirm that the signals are not originating from the starting precursors, but from the clusters (Fig. S14†). The fact that chirality could be achieved only through the presence of DMEB during the synthesis of clusters, and not through post synthetic incubation with DMEB, confirms that the observed optical activity is an intrinsic property of the assemblies and not an induced one. Hence, the nanostructures are classified as intrinsically chiral cluster assemblies. The stability of the aggregate was examined by sonication. It was observed that the aggregates remained stable despite sonication, as evident from the retention of the absorption and emission profiles before and after the process (Fig. S15a and b†).

**Fig. 5:**
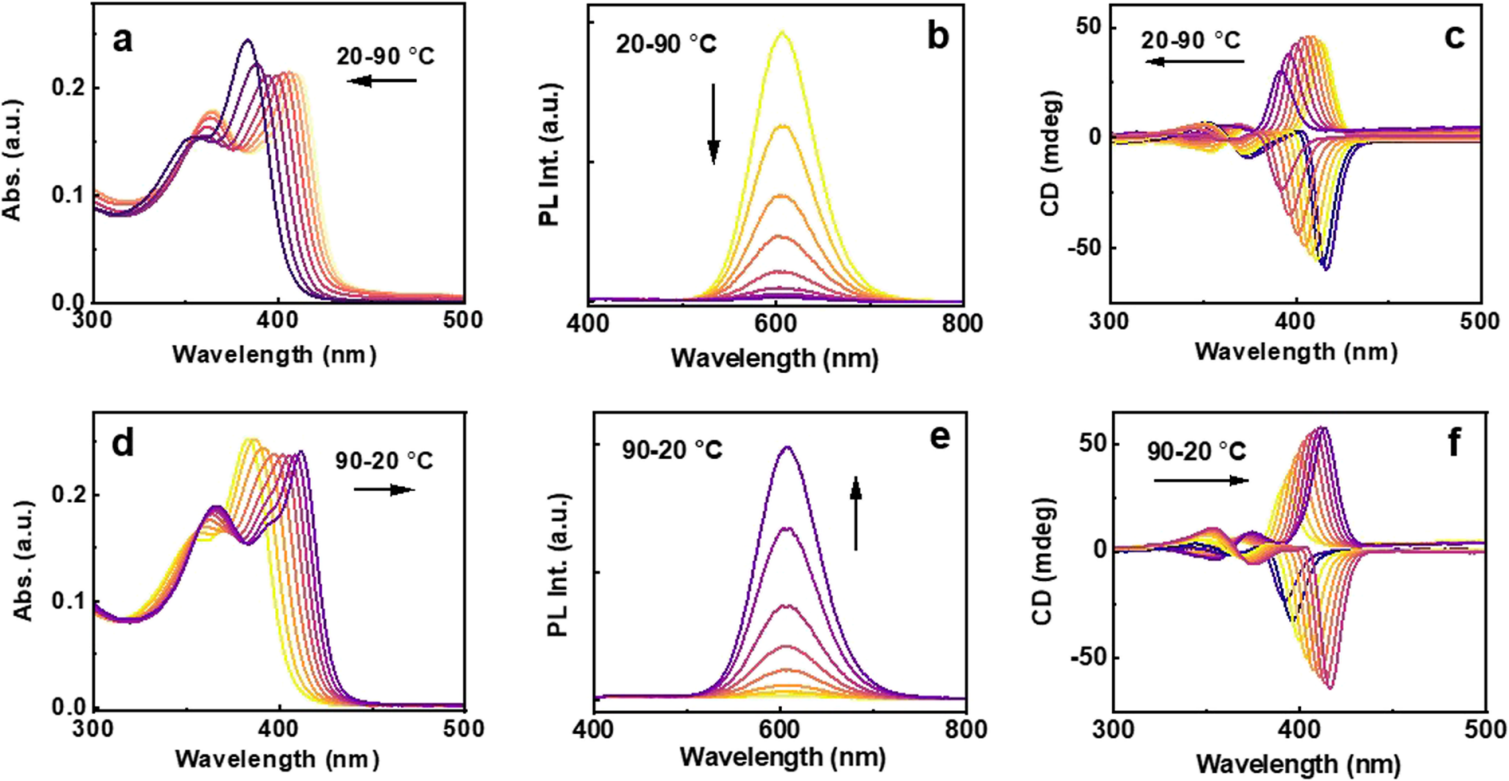
(a) and (d) UV-visible, (b) and (e) luminescence and (c) and (f) CD spectral responses with (a)–(c) a gradual increase in temperature from 20 °C to 90 °C, and (d)–(f) upon cooling back to 20 °C.

To gain deeper insights into the thermal behavior of the clusters, and to understand the stability and structural changes associated with the clusters in the presence of DMEB, temperature dependent investigations were carried out. The diluted solution of the clusters was subjected to heating from 20 °C to 90 °C, followed by cooling back to 20 °C in 10 °C intervals. A gradual hypsochromic shift in the absorption was the notable signature with increasing temperature (Fig. 5a). In accordance with the absorption, the CD spectrum showed a noticeable blue shift, accompanied by a reduction in signal intensity at higher temperatures (Fig. 5c). The blue-shifted absorption and CD profile indicate a widening of electronic bands, likely due to the breaking of the non-bonded interactions at a higher temperature, resulting in an altered energy gap between the HOMO and LUMO. A gradual quenching in luminescence was observed during heating (Fig. 5b). The spectral features could be regained fully upon cooling down the solution to room temperature (Fig. 5d–f) emphasizing that the observed changes are reversible. A structural change likely prompted by the breaking down of the aggregates occurred at elevated temperature. As the temperature decreases, thermal energy diminishes, enabling the structure to revert to its original configuration. The absorption and CD could be switched by varying the temperature, and the cycle could be continued back and forth multiple times proving the robustness of the chiral cluster assembly.

**Fig. 6:**
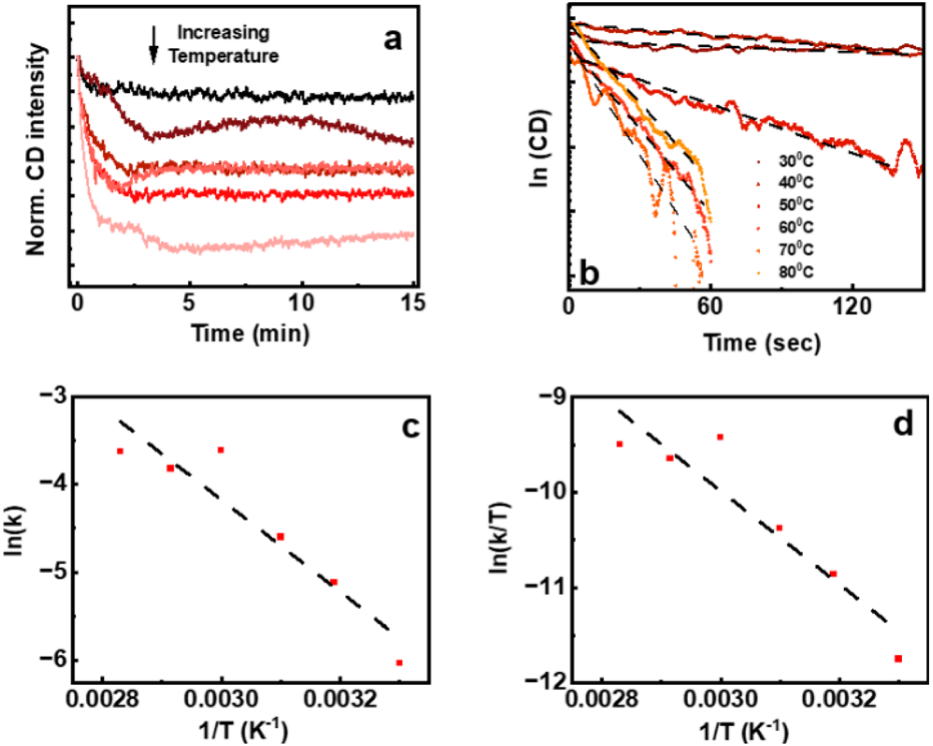
(a) CD response of enantiomerically pure cluster solution at a constant wavelength over a time span of 15 min. The (b) ln(CD) *vs.* time plot, (c) Arrhenius plot and (d) Eyring plot.

To further understand the dynamics of the aggregation, the associated thermodynamic parameters such as changes in reaction rate constant (*k*), activation energy (*E*_a_), entropy (Δ*S*), enthalpy (Δ*H*), and Gibbs free energy (Δ*G*) across various temperatures were calculated. The CD response at different temperatures at a constant wavelength over a time span of 15 min was monitored (Fig. 6a). The plot of ln(CD) against time revealed a linear relationship with a negative slope, affirming the first-order kinetics of the aggregation process (Fig. 6b).^48–50^ However, the complexity of the aggregated system led to a minor deviation from linearity. Subsequently, rate constants at various temperatures were calculated and the corresponding logarithm of the rate constant (ln(*K*)) was plotted against the reciprocal of temperature (1/*T*).^51,52^ The plot was subjected to fitting with the Arrhenius equation (eqn (S1)† and Fig. 6c), allowing a deeper examination of the temperature dependency on the thermodynamic parameters (eqn (S2)†).^53^ Likewise, the natural logarithm of (*K*/*T*) was plotted against the reciprocal of temperature, and the plot was fitted with the Eyring equation (eqn (S3)† and Fig. 6d) facilitating an investigation into the temperature dependence of the thermodynamic parameters (eqn (S4)†).

Based on the Arrhenius plot depicted in Fig. 6c, the activation energy was calculated to be 10 kcal mol^−1^ which is lower than the cohesive energy of Au clusters.^54^ The calculated thermodynamic parameters presented in Table 1 indicate a more negative entropy with increasing temperature. This trend suggests an inverse correlation between temperature elevations and the aggregation process. This indicates that higher temperatures encourage the breakdown of aggregates as observed by the photophysical and chiroptical features, leading to a decrease in entropy. Subsequently, following an increase in temperature, an evident increase in Gibbs free energy suggests the instability of the system at elevated temperatures. This positive Gibbs free energy implies a non-spontaneous process under the stated conditions. However, this non-spontaneous behaviour is amplified by the elevated temperature, as the process is less likely to occur without external intervention. Such an intervention becomes necessary due to factors like unfavourable enthalpy or entropy changes. Similar to the Arrhenius equation, we utilized the Eyring equation to derive parameters, aiming for increased accuracy in our calculations. This is further demonstrated in eqn (S3), (S4) and Table S2.† Upon applying Eyring's equation and examining Fig. 6d, Δ*H* and Δ*S* were determined to be 9.77 kcal mol^−1^ and −2.93 cal mol^−1^ K^−1^. Table S2† contains a detailed list of the calculated thermodynamic parameters at various temperatures. Consequently, it was observed that these thermodynamic parameters aligned well with the values derived from the Arrhenius equation.

**Table 1:** Calculated thermodynamic characteristics using the Arrhenius equation

Temperature (K)	Δ*S* (cal K^−1^ mol^−1^)	Δ*H* (kcal mol^−1^)	Δ*G* (kcal mol^−1^)
303	−2.79	9.81	10.65
313	−2.86	9.79	10.68
323	−2.92	9.77	10.71
333	−2.98	9.75	10.74
343	−3.04	9.73	10.77
353	−3.10	9.71	10.80

To further understand the structural requirements of the co-surfactant in inducing chirality, control experiments were conducted with 3-mercaptopropanoic acid (MPA) and 16-mercaptohexadecanoic acid (MHDA), structural analogues of MUA with different alkyl chain lengths (C_3_ & C_16_). The formation of non-luminescent achiral clusters was observed with MPA (Fig. S16†), with absorption displaying similar features to Au_4_MUA_4_. In contrast, luminescent clusters were formed with MHDA (Fig. S17†), showing similar absorption features. Subsequently, we attempted the synthesis of intrinsically chiral cluster assemblies in these two systems by incorporating DMEB during synthesis. However, significant chirality was not observed in either case (inset of Fig. S16c and S17c†), suggesting that the structural similarity between MUA and DMEB facilitated by the hydrophobic interaction leading to the co-assembly, plays a crucial role in the induction of optical activity. Therefore, incorporating a chiral additive during the synthesis can be an effective strategy to obtain chiral cluster assemblies; however the choice of suitable additive acts as the crucial deciding factor. While numerous attempts have been reported in this direction, achieving intrinsic chirality has remained a major challenge.^55^ The resemblance of long alkyl chains in DMEB and MUA would facilitate hydrophobic interactions whereas the opposite surface charge can drive electrostatic attraction leading to effective assembly with enhanced performance. To the best of our knowledge, this is the first attempt towards achieving inherently chiral cluster aggregates with enhanced properties through a simple chiral additive method.

## Conclusions

In summary, we have successfully demonstrated the assembly of negatively charged, red-emitting achiral gold nanoclusters in the presence of the enantiomerically pure surfactant, DMEB, at a controlled pH. Excited state investigations revealed that the observed longer lifetime decay has contribution from the triplet state. The chiroptically active assemblies demonstrated enhanced luminescence due to the AIEE effect. The luminescence and the chiral signals of the nanoaggregates were responsive to thermal changes, highlighting the possibility of developing a temperature sensor. Temperature-dependent investigations revealed the thermodynamic parameters for the assembled structures, with calculations performed using both the Arrhenius and Eyring equations. At higher temperatures, the nonbonded interactions responsible for the aggregation partially break down, leading to the decrease in chiroptical properties. Therefore, our endeavor to introduce chirality through the concept of co-assembly with a chiral dopant deepens the fundamental understanding on the ground state chirality and excited state luminescence in cluster assemblies and has the potential to open newer avenues for such research on a wide range of alternate nanomaterials.

## Data availability

The data supporting this article have been included as part of the ESI.†

## Author contributions

J. K. conceived and coordinated the project. C. D. and R. V. N. carried out the experiments. C. D. & J. K. analysed and consolidated the data. C. D. and J. K. prepared the manuscript. All authors have given approval to the final version of the manuscript.

## Conflicts of interest

There are no conflicts to declare.

## Supplementary Material

SC-016-D4SC07227H-s001

SC-016-D4SC07227H-s002
