# Supplementary figures and images for: Intrinsically chiral thermoresponsive assemblies from achiral clusters: enhanced luminescence and optical activity through tailor-made chiral additives
Source: Chem Sci. 2024 Dec 17;16(4):1722–9. doi: 10.1039/d4sc07227h (PMC11665439; doi:10.1039/d4sc07227h)

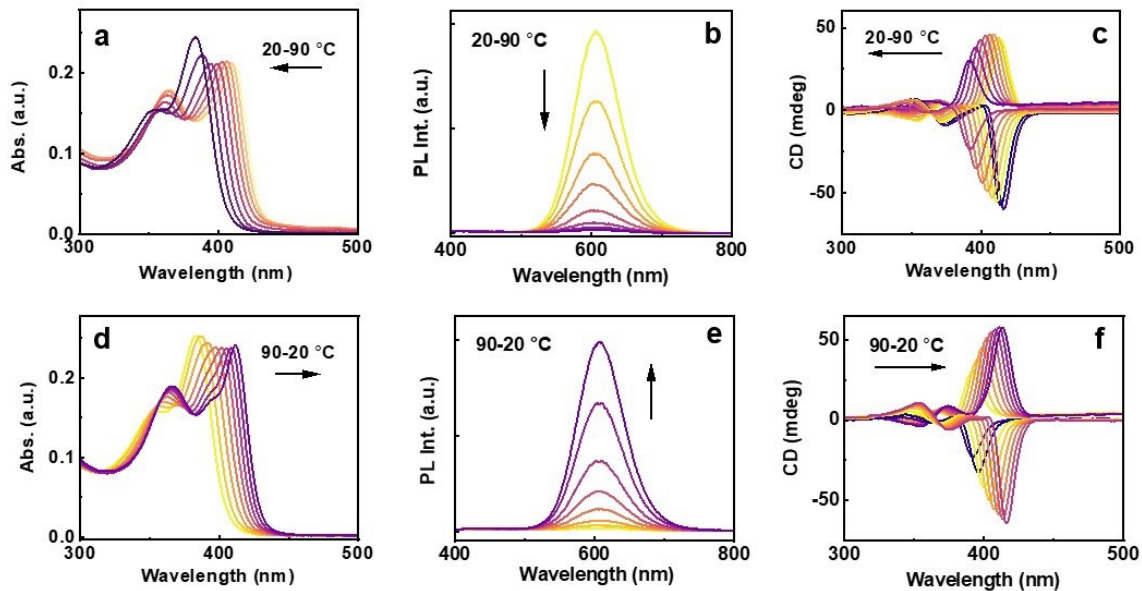

Supplement: SC-016-D4SC07227H-s001 [file SC-016-D4SC07227H-s001.pdf]
