# Supplementary material for: Intrinsically chiral thermoresponsive assemblies from achiral clusters: enhanced luminescence and optical activity through tailor-made chiral additives
Source: Chem Sci. 2024 Dec 17;16(4):1722–9. doi: 10.1039/d4sc07227h (PMC11665439; doi:10.1039/d4sc07227h)
Supplement: SC-016-D4SC07227H-s002 [file SC-016-D4SC07227H-s002.pdf]

Supplementary Information (SI) for Chemical Science.
This journal is © The Royal Society of Chemistry 2024

Intrinsically chiral thermoresponsive assemblies from achiral clusters: enhanced luminescence and optical activity through tailor-made chiral additives

Camelia Dutta,[a] Ragul Vivaz Nataraajan,[a] Jatish Kumar*[a]

Department of Chemistry, Indian Institute of Science Education and Research (IISER) Tirupati, Tirupati – 517619, India.

Email: jatish@iisertirupati.ac.in

Table of Contents

Sl. No.	Content	Page No.
1	Experimental details	S2
2	Fig. S1: MALDI-TOF plot of Au_4 clusters.	S3
3	Fig. S2: XPS survey scan of Au_4 clusters.	S3
4	Fig. S3: TEM images of MUA capped gold nanoclusters	S4
5	Fig. S4: Lifetime plot of clusters in presence and absence of DMEB	S4
6	Fig. S5: CD spectra of MUA capped gold nanoclusters	S4
7	Fig. S6: Zeta potential of clusters in presence and absence of DMEB	S5
8	Fig. S7: Titration experiment of the clusters against (-)-DMEB.	S5
9	Fig. S8: FT-IR spectrum of DMEB and clusters in presence of DMEB	S5
10	Fig. S9: TEM images of the clusters in presence of DMEB	S6
11	Fig. S10: Temperature dependent lifetime in absence of DMEB.	S6
12	Fig. S11: Lifetime versus temperature plot of the chiral aggregates	S7
13	Table S1: Data from the calculations from Boltzmann equation	S7
14	Fig. S12: CD plot of the chiral aggregates synthesized at different pH	S8
15	Fig. S13: Time dependent CD evolution in cluster aggregates	S8
16	Fig. S14: Spectral features for control samples	S9
17	Fig. S15: Spectra of the clusters before and after sonication	S9
18	Fig. S16: Spectra of clusters synthesized with MPA and DMEB	S10
19	Fig. S17: Spectra of clusters synthesized with MHA	S10
20	Thermodynamic parameters calculation	S11
21	Table S2: Table summarizing the thermodynamic parameters	S11

METHODS

Chemicals and materials

Gold(III) chloride trihydrate ($HAuCl_4$), 11-Marcaptoundecanoic acid (MUA), (-)N-dodecyl-N-methylephedrinium bromide [(-)-DMEB], sodium borohydride ($NaBH_4$), sodium hydroxide (NaOH) pellets, N-methylephedmine, bromododecane, diethyl ether were purchased from Sigma-Aldrich. (+)-DMEB was synthesized in laboratory. All the glass wares were washed with aqua regia (HCl: HNO3 = 3:1), milli-Q water, and acetone and were dried in oven at 60 $^{\circ}$C. Milli-Q water was consistently utilized throughout the entirety of the study.

Characterization

UV-visible absorption, photoluminescence, and CD spectrum were collected by using Cary UV-vis Multicell Peltier, JASCO spectrofluorometer FP-8500, and JASCO J-1500 CD spectrometer, respectively. Zeta potential measurements were conducted using the Litesizer 500. FT-IR spectra was collected by BRUKER ECD-ART instrument. Lifetime measurements were carried out on Edinburg FLS-1000 fluorescence spectrometer, with an excitation laser of 375 nm. Absolute quantum yield measurements were acquired employing the integrating sphere within an Edinburg FLS-1000 instrument. XPS measurements were carried out using an ESCA Plus spectrometer (Omicron Nanotechnology Ltd., Germany) using a Mg Kα source. TEM images were captured using FEI Tecnai G2 20 S-twin electron microscope with an acceleration voltage of 200 kV.

Synthesis of (+)-DMEB

(+)-DMEB was prepared by refluxing (1S,2R)-(+)-N-methylephedamine (1.0 g, 5.6 mmoles) and 1-bromododecane (1.1 equivalent) in ethanol at 85 $^{\circ}$C for 48 hours with vigorous stirring. Subsequently, the solvent was evaporated under vacuum, and the resulting crude product was collected. This crude product was further purified by washing with ethylacetate and hexane to yield the purified product.

Synthesis of Au_4MUA_4

MUA-capped AuNCs were synthesized using a previously reported method with minor modifications. A basic solution of MUA was prepared by adding 75 µL of 1M NaOH to 10 mL of Milli-Q water containing 6.6 mg of MUA. Subsequently, 10 µL of 1 M $HAuCl_4$ was added to the prepared basic solution of MUA, and the resulting mixture was stirred for 24 h in the dark at room temperature. The product was then centrifuged at 10,000 rpm for 10 min followed by dialysis for 48 h, and the purified Au_4MUA_4 containing solution was stored in the refrigerator at 4 $^{\circ}$C.

Synthesis of Au_4MUA_4 in presence of DMEB

For synthesizing the clusters, basic solution of MUA was prepared by adding 50µL NaOH and 6.6 mg MUA into 10 mL of water. The mixture was kept under stirring at 500 rpm for half an hour. Subsequently, 5µL of 0.05 M (+)/(-)-DMEB and 10 µL of 1 M $HAuCl_4$ were added into the solution, and the solution was kept under stirring for 48 h at dark. The resultant solution turned turbid and the purification steps were performed to obtain pure MUA capped chiral Au_4 nanoclusters. Bigger particles and excess reactants were removed by centrifuging the solution

at 10,000 rpm for 20 min and the supernatant was collected. Subsequently, the clusters were purified by dialysis using dialysis membrane of average diameter 21.5 mm for 24 h. The purified cluster solution was stored in refrigerator at 4 °C.

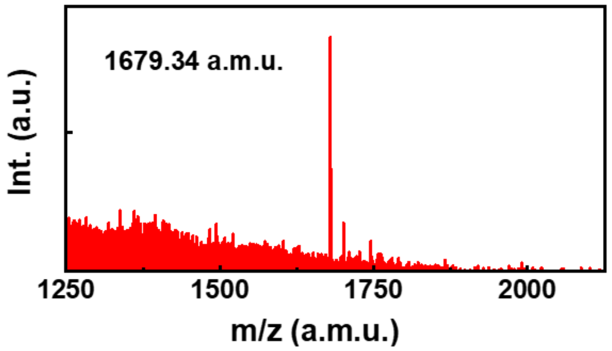

Figure S1. MALDI-TOF spectra of $Au_4(MUA)_4$ clusters.

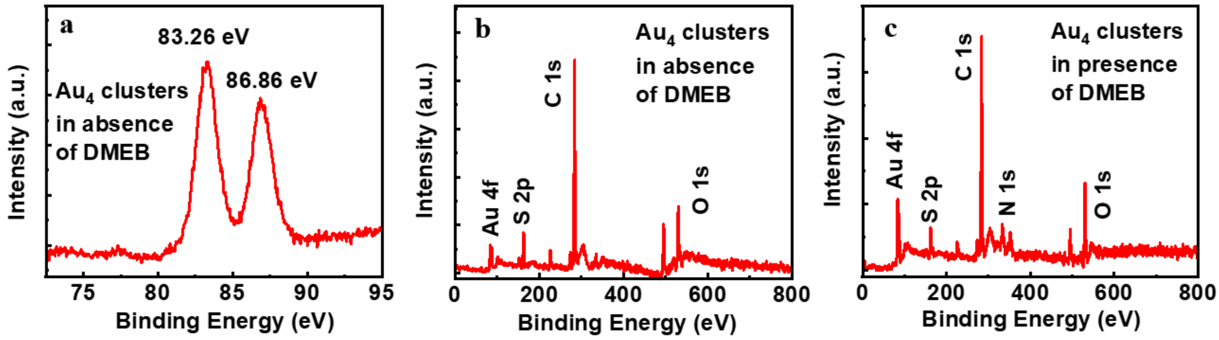

Figure S2. (a) XPS survey of $Au_4(MUA)_4$ clusters corresponding to Au 4f. The XPS spectrum of Au_4 clusters in (b) absence and (c) presence of DMEB.

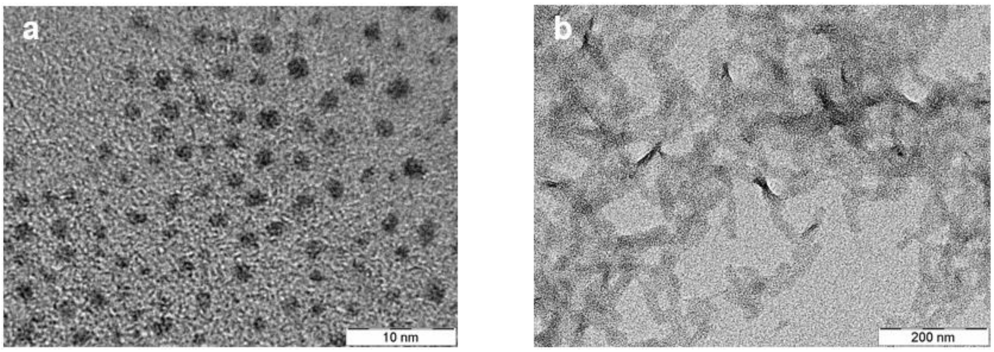

Figure S3. TEM images of MUA capped gold clusters synthesized in the (a) absence and (b) presence of the base.

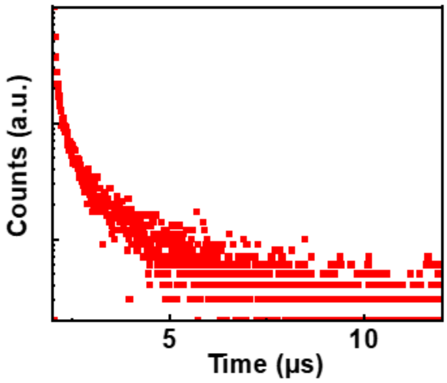

Figure S4. Lifetime decay plot of MUA capped Au_4 clusters.

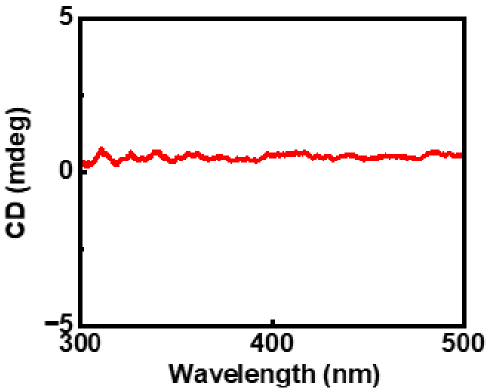

Figure S5. CD spectrum of MUA capped Au_4 clusters dispersed in water.

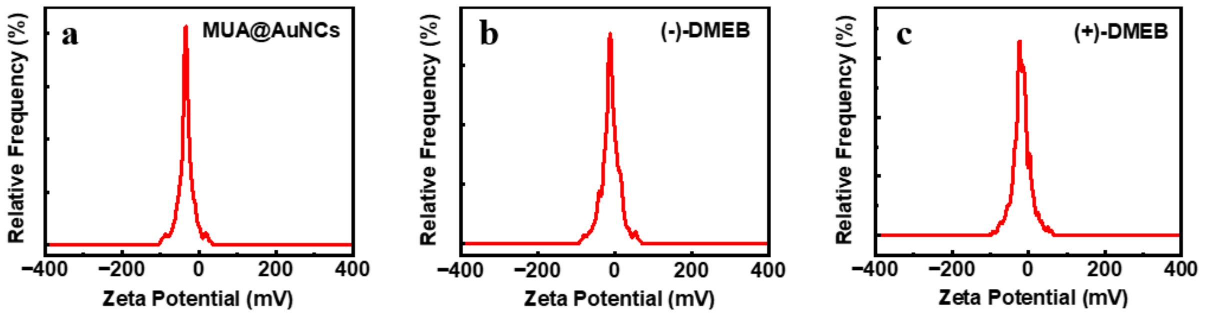

Figure S6. Zeta potential plot of the clusters in (a) absence and (b,c) presence of (b) (-)-DMEB and (c) (+)-DMEB.

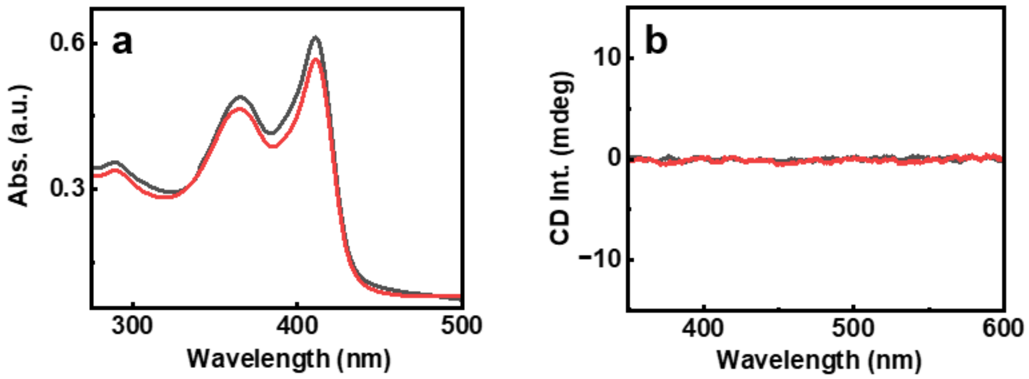

Figure S7. (a) Absorption and (b) CD spectra of MUA clusters before (black trace) and after (red trace) titrating it against (-)-DMEB.

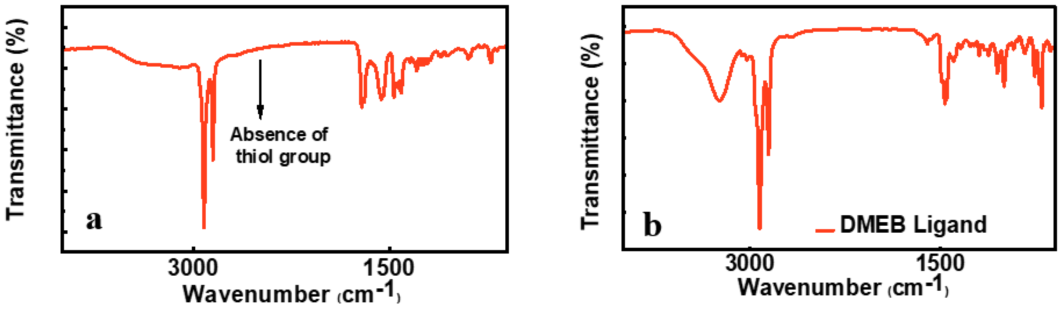

Figure S8. FT-IR spectra of the (a) Au_4 clusters in presence of DMEB and (b) pure DMEB.

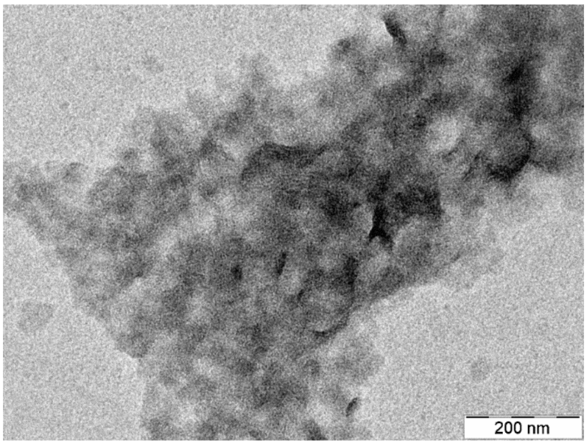

Figure S9. TEM images of the clusters in presence of DMEB.

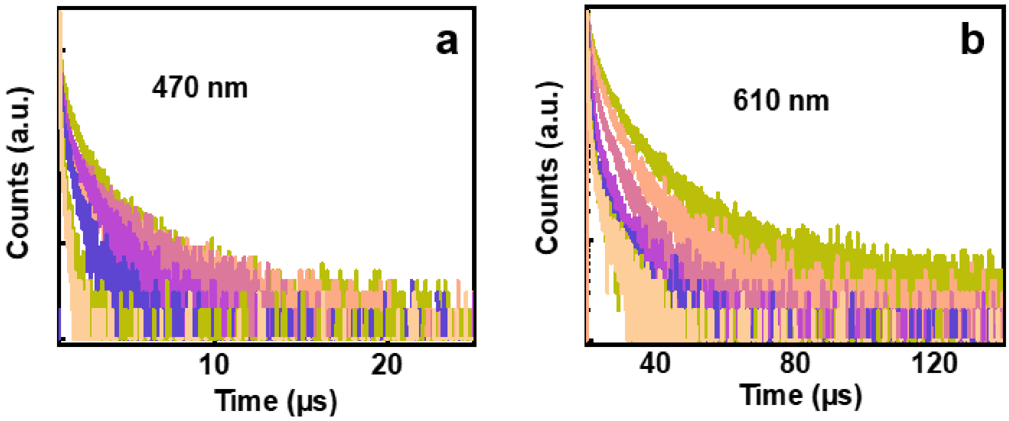

Figure S10. Temperature dependent lifetime plot of pure MUA gold clusters at (a) 470 nm and (b) 610 nm wavelength.

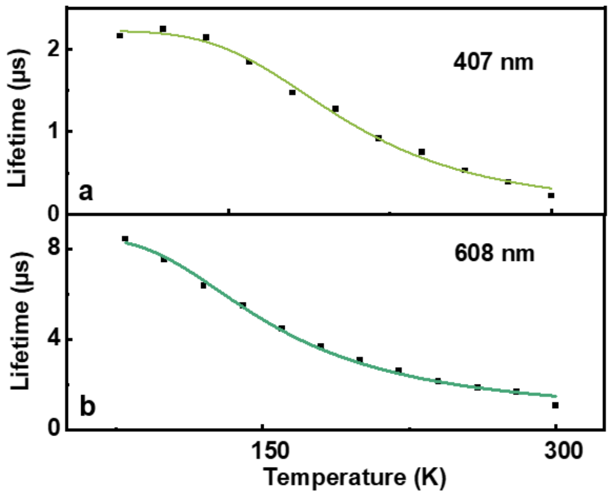

Figure S11. Lifetime versus temperature plot of the chiral aggregates in MCS mode corresponding to (a) 407 and (c) 608 nm PL emission.

Lifetime versus temperature plot of the chiral aggregates corresponding to higher and lower emission yielded a sigmoidal plot indicating the possibility of reverse inter-system crossing (RISC) at room temperature. To investigate the emission mechanism further, the data were fitted using the Boltzmann equation derived for TADF model (Figure S11). The fitting showed agreement with the theoretical model, revealing a singlet-triplet energy gap (ΔE_{ST}) less than 0.37 eV. This small energy gap would facilitate efficient RISC, opening the possibility of delayed fluorescence at both emission wavelengths at room temperature (Table S1).

The calculated ΔE_{ST}, singlet decay rate constant ($k(S_1)$), triplet decay rate constant ($k(S_1)$) and singlet lifetime ($\tau(S_1)$), triplet lifetime e($\tau(T_1)$) are listed below in Table S1.

Table R1. Calculated thermodynamic characteristics using Boltzmann equation.

**Emission Wavelength (nm)**	**Fitting (%)**	**ΔE_{ST} (eV)**	**$k(S_1)$ ($\times 10^6$ s^{-1})**	**$k(T_1)$ ($\times 10^6$ s^{-1})**	**$\tau(T_1)$ (μs)**	**$\tau(S_1)$ (ns)**
**407**	99.3	0.1	438.6	45.045	2.2	2.28
**608**	99.4	0.05	11.79	11.78	8.49	84.82

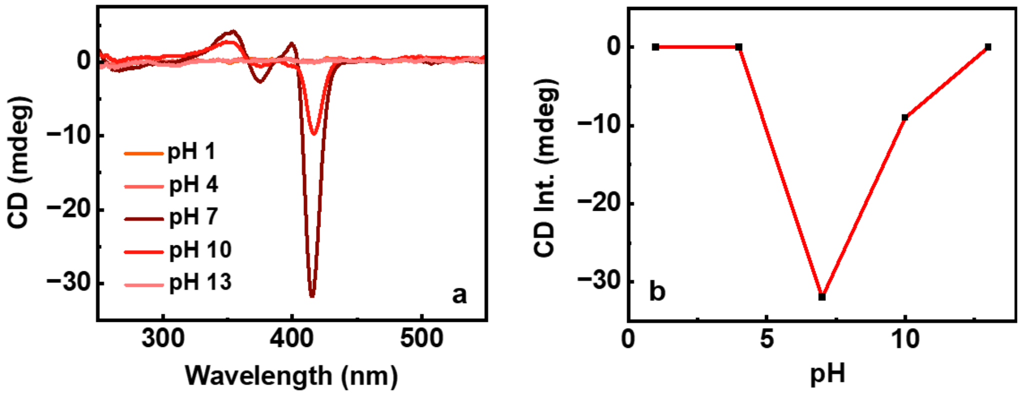

Figure S12. (a) pH dependent CD spectra of the clusters synthesized in presence of DMEB. (b) CD intensity versus pH plot.

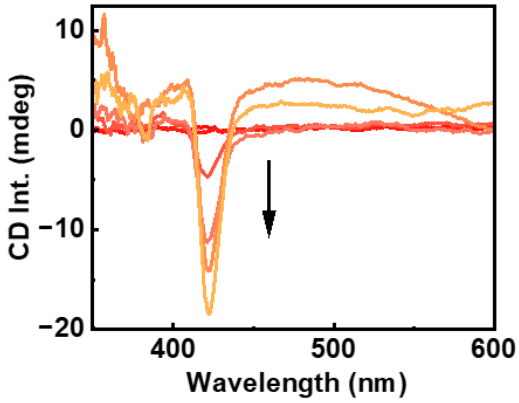

Figure S13. Evolution of CD signal at varying reaction timings in presence of DMEB.

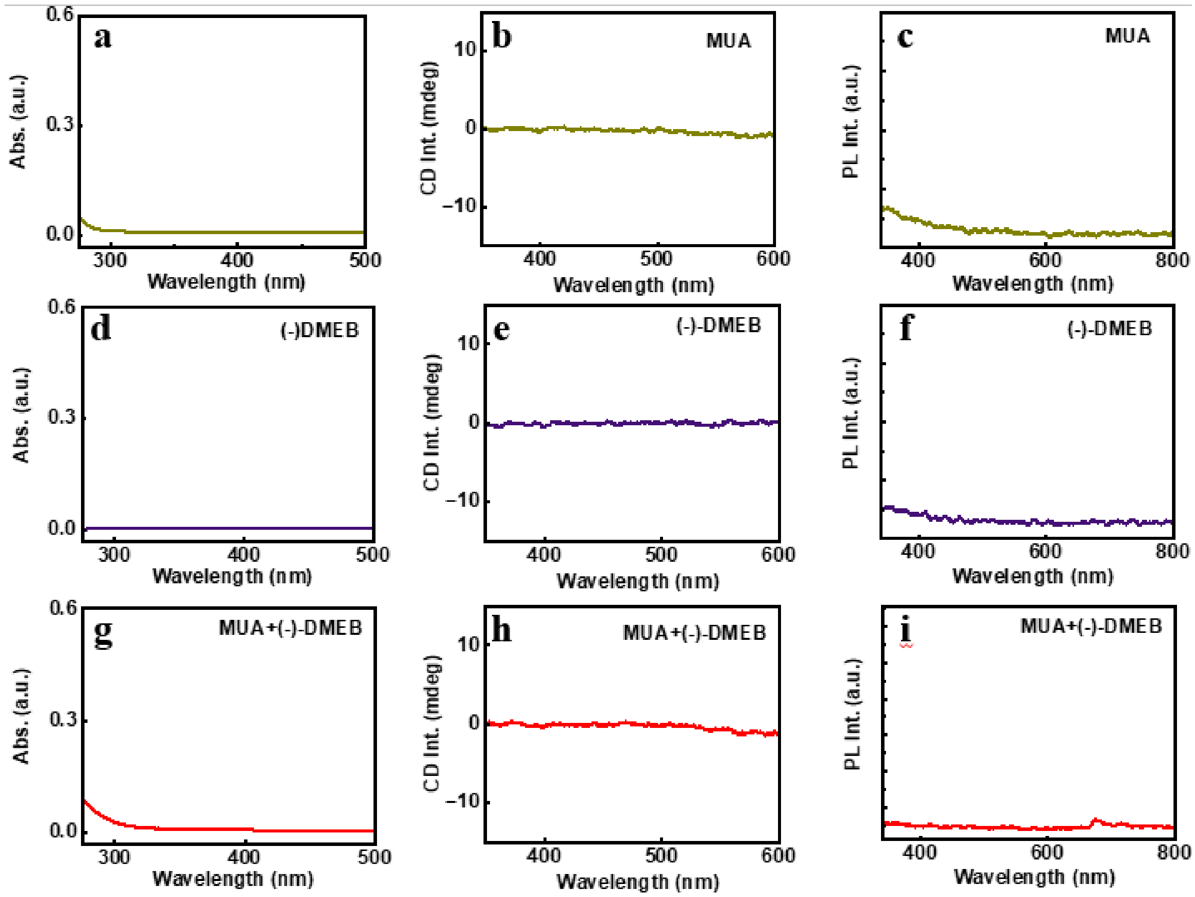

Figure S14. (a,d,g) Absorption, (b,e,h) PL and (c,f,i) CD spectra of (a,b,c) only MUA in presence of base, (d,e,f) only DMEB in basic medium and (g,h,i) a mixture of MUA and DMEB at a basic pH.

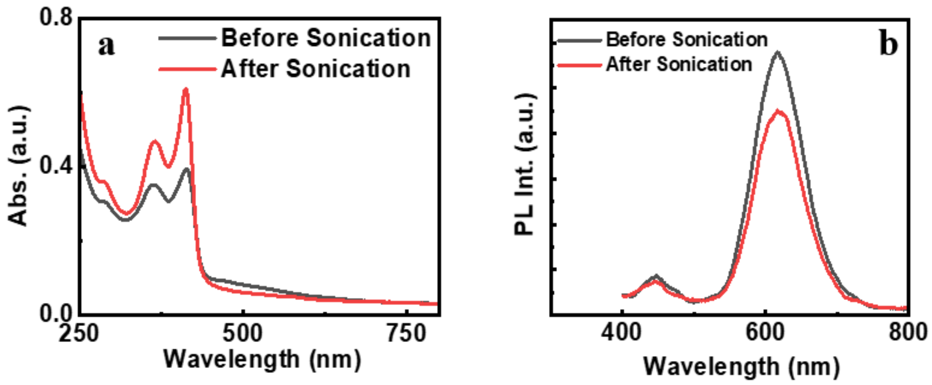

Figure S15. (a) Absorption and (b) PL spectra of the clusters before and after sonication.

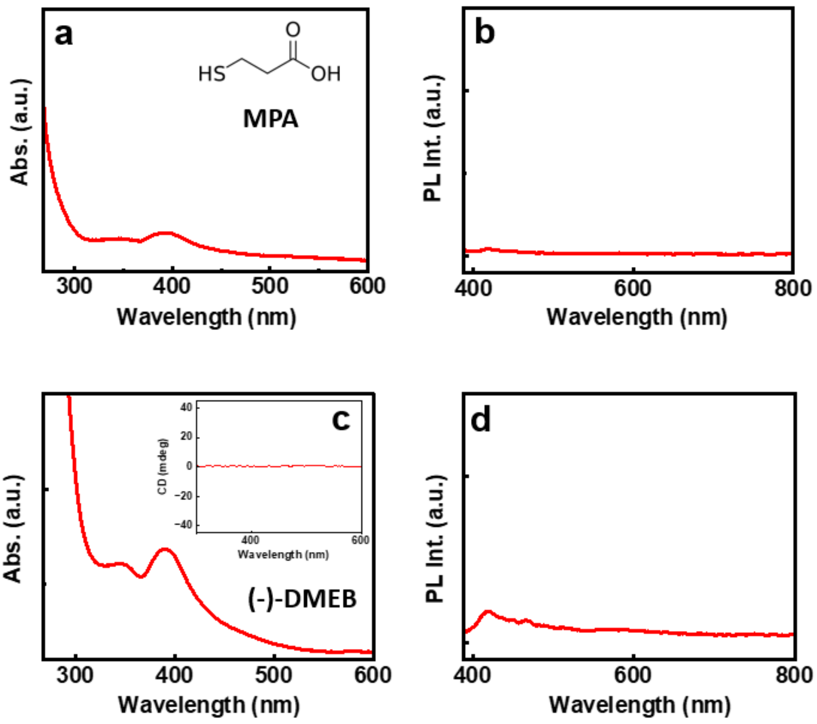

Figure S16. (a,c) absorption and (b,d) emission spectra of the clusters synthesized using 3-mercaptopropanoic acid as a capping ligand in (a,b) absence and (c,d) presence of (-)-DMEB. Inset in (c) shows the corresponding CD spectra in presence of (-)-DMEB.

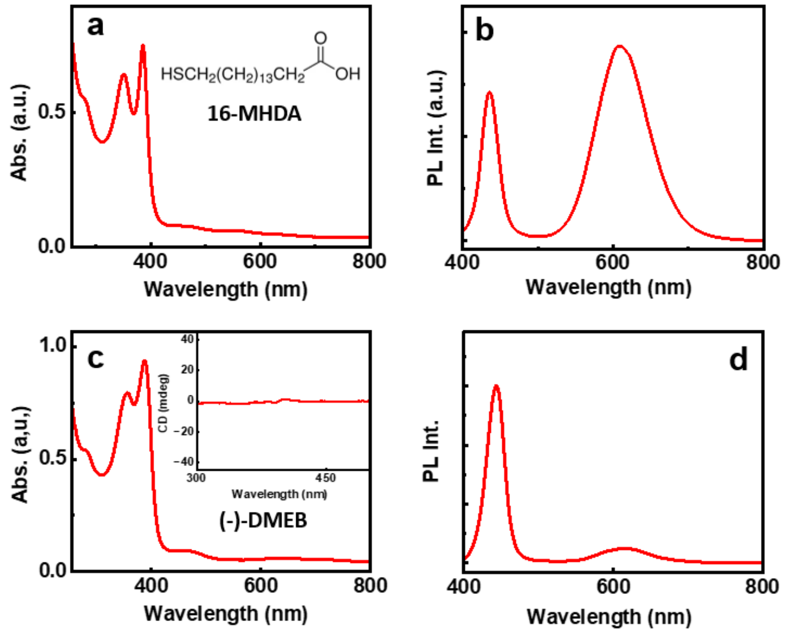

Figure S17. (a,c) absorption and (b,d) emission spectra of the clusters synthesized using 16-mercaptohexadecanoic acid as a capping ligand in (a,b) absence and (c,d) presence of (-)-DMEB. Inset in (c) shows the corresponding CD spectra in presence of (-)-DMEB.

Thermodynamic Parameters Calculation:

Arrhenius Equation: The temperature dependence on the thermodynamic parameters has been calculated by utilizing the following equations, where R and A are Universal gas constant, and pre-exponential factor, respectively.

$$k = Ae^{-\frac{E_a}{RT}}$$

$$ln\, ln\,(k) \ = \frac{-E_a}{R} \times \frac{1}{T} + lnA \qquad \text{.................. (S1)}$$

$E_a, \Delta H, \Delta S,$ and ΔG were determined from figure 5c using the following equations 2a, 2b, 2c, and 2d, respectively where k_B and, h are Boltzmann constant, and Plank's constant, respectively.

$$E_a = -R \times slope \qquad \text{................... (S2a)}$$

$$\Delta H = E_a - RT \qquad \text{................... (S2b)}$$

$$\Delta S = R(intercept - ln\frac{ek_BT}{h}) \qquad \text{................. (S2c)}$$

$$\Delta G = \Delta H - T\Delta S \qquad \text{................... (S2d)}$$

Eyring Equation:

$$ln\frac{k}{T} = \frac{-\Delta H}{R} \times \frac{1}{T} + ln\frac{k_B}{T} + \frac{\Delta S}{R} \qquad \text{..................... (S3)}$$

$E_a, \Delta H, \Delta S,$ and ΔG were determined from figure 5d using the following equations S4a, S4b, S4c, and S4d, respectively.

$$E_a = \Delta H + RT \qquad \text{...................... (S4a)}$$

$$\Delta H = -R \times slope \qquad \text{...................... (S4b)}$$

$$\Delta S = R(intercept - ln\frac{k_B}{T}) \qquad \text{...................... (S4c)}$$

$$\Delta G = \Delta H - T\Delta S \qquad \text{...................... (S4d)}$$

Table S2. Calculated thermodynamic parameters using Eyring's equation.

Temperature (K)	E_a (Kcal mol^{-1})	ΔG (Kcal mol^{-1})
303	10.38	10.66
313	10.40	10.69
323	10.42	10.72
333	10.44	10.75
343	10.46	10.78
353	10.48	10.81
